# Application of *N*-Dodecyl l-Peptide to Enhance Serum Stability while Maintaining Inhibitory Effects on Myometrial Contractions Ex Vivo

**DOI:** 10.3390/molecules24224141

**Published:** 2019-11-15

**Authors:** Julien Poupart, Xin Hou, Sylvain Chemtob, William D. Lubell

**Affiliations:** 1 Département de Chimie, Pavillon Roger Gaudry, Université de Montréal, CP 6128 and Succursale Centre-ville, Montréal, QC H3C 3J7, Canada; julien.poupart@umontreal.ca; 2 Centre de recherches du Centre Hospitalier Universitaire Sainte-Justine, Montréal City, QC H3T 1C5, Canada; xin.hou82@gmail.com (X.H.); sylvain.chemtob@gmail.com (S.C.)

**Keywords:** bioconjugation, lipidation, prostaglandin F_2α_, preterm labor, myometrium contractions

## Abstract

*N*-Alkylation and *N*-acylation of the prostaglandin-F_2α_ allosteric modulator l-PDC31 were performed to install various alkyl, PEG and isoprenoid groups onto the l-enantiomer of the peptide. Among the different bio-conjugates studied, the *N*-dodecyl analog reduced prostaglandin-F_2α_-induced mouse myometrium contractions ex vivo. Furthermore, *N*-dodecyl-l-PDC31 exhibited improved stability in a mouse serum assay, likely due to protection from protease degradation by the lipid chain.

## 1. Introduction

Preterm labor is a challenging contemporary target of modern medicinal chemistry. Preterm birth (PTB), defined as less than 37 weeks of gestational age, accounts for 5–13% of all births [1]. Responsible for an increased risk of neonatal and infantile mortality [2] PTB is associated with various other complications, including developmental disorders [3], vision and hearing impairments [4,5], and chronic metabolic conditions [6]. Since 2012, PTB has been the single most expensive medical urgency per patient in the United States [7], in part due to a lack of so-called tocolytic drugs that can effectively and safely delay delivery.

In less affluent countries, PTB is more problematic: >60% of PTBs occur in Africa and South-Asia. Ineffective healthcare to treat mother and child in less affluent countries is associated with a higher percentage of neonatal and infantile mortality: 90% for very preterm babies of <28 weeks. In developed countries, <10% of newborns suffering similar PTB conditions experience the same fate [7].

The currently available medications, including the oxytocin receptor antagonist Atosiban [8], calcium channel blockers [9], β_2_ agonists [10], a myosin light chain inhibitor [11], and prostaglandin-endoperoxide synthase inhibitors [12], all rarely delay labor for >48 h [13,14] and many have risks of serious side effects for both mother and infant. New approaches exploring different pathways to tocolytic agents are essential for the development of more effective treatments with minimal side effects. For ideal distribution to third-world countries, such tocolytic agents would be produced cost-effectively.

The prostaglandin F2α receptor (FP) is a promising target for delaying labor because of its relevant involvement in parturition. At the beginning of labor and preterm labor, FP expression increases [15,16]. Moreover, FP-knockout mice do not go into labor, even when induced by oxytocin [17]. Targeting FP, the tocolytic prototype PDC31 is currently undergoing clinical trials [18,19]. In a phase Ib clinical trial of women with primary dysmenorrhea, PDC31 was demonstrated to be safe in reducing intrauterine pressure and pain associated with excessive uterine contractility [20].

The development of PDC31 originated from a library of sequences based on the second extracellular loop of FP. Short lead peptides were analyzed initially using *in vitro* and ex vivo analyses [19]. The lead peptide PDC113 (H-ile-leu-Gly-his-arg-asp-tyr-lys-OH, small letters refer to D amino acids) was identified as an active FP inhibitor [18]. Replacement of the arginine residue with citrulline yielded PDC31 (H-ile-leu-Gly-his-cit-asp-tyr-lys-OH, cit = d-citrulline), which exhibited notable efficacy (>85%) and potency (IC_50_ = 13 nM) [18]. Selectively modulating the downstream signaling of FP, the mechanism of action of PDC31 is suggested to involve allosteric inhibition at a site different from the “othosteric”, native ligand-binding site [19,21].

The relatively high price of d-amino acid building blocks can influence the cost of production of PDC31, which is composed of seven of d-amino acids including d-citrulline. The l-amino acid version of PDC31 has been observed to exhibit some antagonist activity but failed to do so in a myometrial tissue-based assay, probably owing to the relatively low metabolic stability of linear l-peptides. To improve the metabolic stability of l-PDC31, the peptide *N*-terminal has now been modified with lipid chains. Previously, *N*-acetylation had abolished the activity of PDC113 analogs. Thus, instead of acylation, a strategy was pursued to maintain the basic nitrogen by alkylation with different hydrophobic moieties: linear hydrocarbons, PEG and farnesyl chains.

Polyethylene glycol grafts onto polyamide structures have demonstrated success for improving pharmacodynamic properties, particularly duration of action [22,23]. Protein PEGylation has enhanced water solubility, prolonged half-life, improved metabolic stability and diminished immunogenicity compared to the unmodified molecule. Polyethylene glycol chains are often employed as a distribution of related lengths grafted indiscriminately on various residues [24,25]. Successful examples on which polyethylene glycol grafts enhanced potency and duration of action of larger proteins include Pegasys© for hepatitis C [26] and Pegvisomant for the treatment of acromegaly (Somavert©) [27]. Moreover, modification of the glucagon-like peptide 1 (GLP-1)-based drug Liraglutide^®^, which features a C16 lipid chain responsible, in part, for its longer duration of action, by adding a PEG-like spacer gave Semaglutide^®^, which can be administered once weekly and is used in the treatment of type 2 diabetes and obesity [28,29]. Lipid attachment has also been used to improve metabolic stability and lipophilicity [30,31] to enhance the stability of larger peptides, as well as to improve their specificity and association with membranes [32]. Lipopeptide natural products, such as daptomycin and related antibiotics have exhibited promising activity against Gram-positive pathogens [33]. Lipid conjugation has also been applied to smaller peptides. For example, Hruby and co-workers have attached hexanoyl and decanoyl chains onto α-Melanocyte Stimulating Hormone (αMSH) [34] analogs to obtain equipotent conjugates with prolonged duration of action [31,35]. In contrast, longer chains (palmitic and myristic acids) resulted in less potent molecules, probably because of the increased lipophilicity [31,35]. Peptide-based vaccines have been created with lipid components that act as adjuvants [36]. Amphiphiles, peptides bearing both a hydrophobic lipid chain and a charged moiety have been showed to aggregate in liposomes that can act as carriers for targeted drug delivery [37,38]. The strategy called reversible aqueous lipidation (REAL) has been used to improve the bioavailability of prodrugs that release the active molecule in the organism and applied to facilitate oral delivery of small peptides [30]. Metabolic stability and membrane permeability were improved by acylation of small peptides with a palmitoyl chain that facilitated anchoring in membranes and may enhance peptide folding [39].

Prenylation is an important post-translational protein and peptide modification, which enhances typically cell membrane association [40,41,42,43]. Farnesyl transferase enzymes introduce farnesyl subunits that may serve to anchor the modified protein or peptide in the membrane. Disruption of prenylation has been suggested to play an important role in cancer because of the significance of membrane-associated proteins in the cellular life cycle [44]. Prenylation by prenyl transferases of small molecules, such as prenylflavonoids may also influence biological effects by favoring membrane association [45].

Seeking to improve the pharmacokinetic properties of l-PDC31, a variety of analogs were developed. Different *N*-alkyl terminal grafts were introduced by solid phase methods featuring reductive amination and sulfonamide alkylation. Among the analogs evaluated in a murine ex vivo myometrium contraction assay, the best was selected for study in a mouse serum stability test and in an in vivo PTB model.

## 2. Results

### 2.1. Chemistry

**Figure 1:**
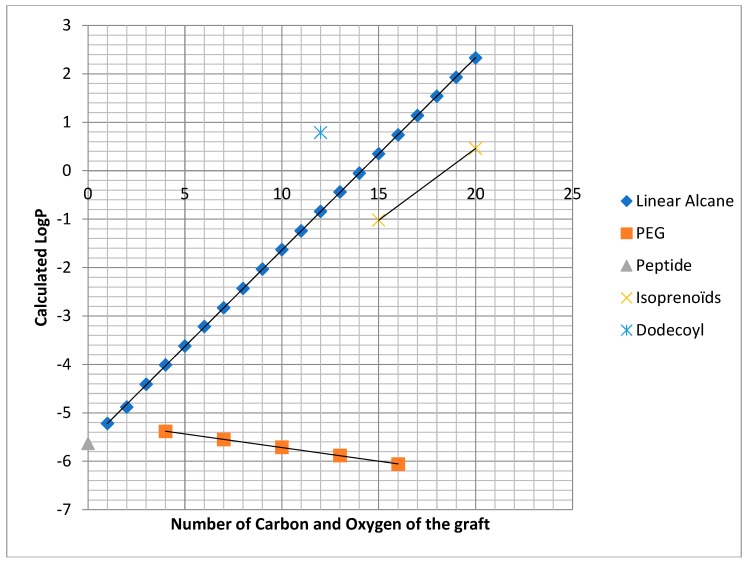
*c*LogP of l-PDC31 analogs relative to the number of heavy atoms present in their grafts.

The influence of the different hydrophobic chains was initially evaluated by a computational model in which logP values were predicted using the HyperChem software [46,47] and compared with PDC-31 (Figure 1). Small PEG chains of 4 to 16 atoms had limited impact and lowered the logP of the peptide analogs. Alternatively, the addition of *n*-alkyl chains caused a notable increase with a nearly linear correlation in log P with increasing carbon length.

The branched prenyl group increased logP with the effect appearing to be related to the longest linear chain. For example, the 15-carbon farnesyl chain produced a peptide derivative that was predicted to have a logP as that of the peptide possessing a linear 12-carbon alkyl chain. Similarly, the branched 20-carbon geranyl-geranyl group exhibited a logP lower than its 16-carbon linear alkyl chain equivalent.

Based on the above predictions, a set of analogs of the l-peptide variant of PDC31 were targeted possessing *N*-terminal chains consisting of 6-, 11-, 12-, 13- and 18-carbon linear alkyl, tetra-ethylene glycol [48] and farnesyl groups. In addition, *N*-dodeconyl-l-PDC31 (**8**) was synthesized as an acyl control using lauric acid.

**Scheme 1:**
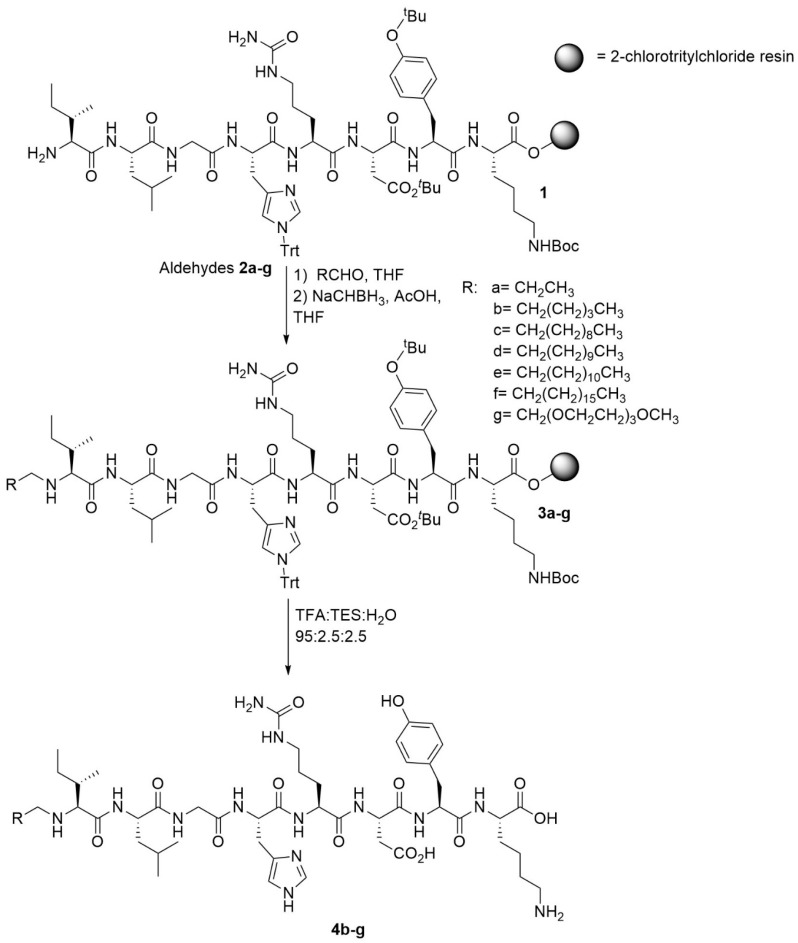
Synthesis of peptides **l**–**4b**–4**g**.

Linear sequences of l- and d-PDC31 linked to 2-chlorotrityl resin (l- and d-**1**) were obtained by solid phase peptide synthesis using standard Fmoc/t-Bu protocols [49]. Peptide resin l-**1** was treated with the appropriate aldehyde **2** (Scheme 1). The imine resin was washed to remove excess aldehyde, and reductive amination was performed using sodium cyanoborohydride to provide secondary amine resin **3**. Removal of the protecting groups and resin cleavage were achieved using a cocktail of 95:2.5:2.5 TFA:TES:H_2_O to provide *N*-alkyl- and *N*-PEG-grafted peptides **4**, which were purified by RP-HPLC (Table 1). Except for peptide **4e**, bearing the 18-carbon alkyl chain, peptides **4** were all soluble in water. Peptide **4f** required a small amount of DMSO to completely dissolve. An inseparable mixture of mono- and bis-*N*-alkyl peptides was isolated by HPLC from reaction with aldehyde of shorter chain length.

**Scheme 2:**
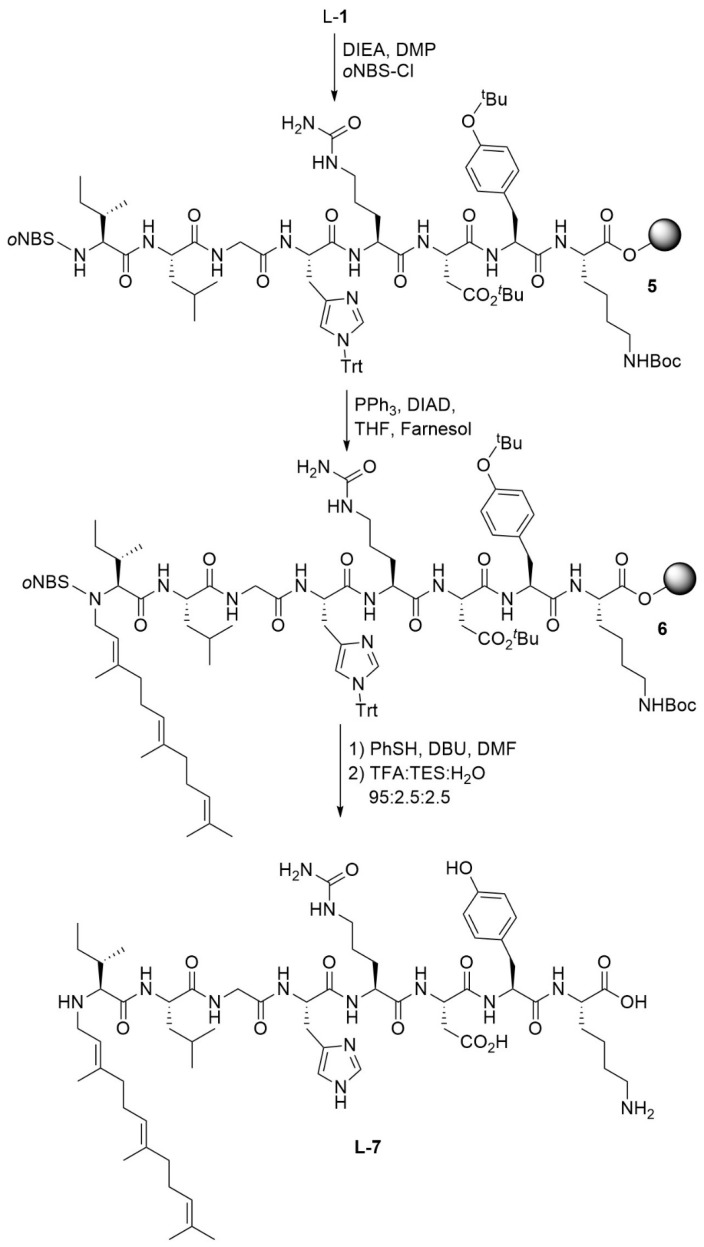
Synthesis of *N*-farnesyl-l-PDC31 (**l–7**).

Farnesyl peptide **7** was synthesized by a route featuring sulfonylation followed by alkylation with farnesol under Mitsunobu conditions. Resin l-**1** was swollen in DMF and reacted with *ortho*-nitrobenzenesulfonyl (*o*NBS) chloride and di-*iso*-propylethylamine (Scheme 2). With the *o*NBS group installed, sulfonamide resin **5** was treated with di-*iso*-propyl azodicarboxylate (DIAD), triphenylphosphine and farnesol. *N*-Farnesyl peptide resin **6** was provided in 55% conversion as demonstrated by LC-MS analysis of material after resin cleavage [50,51,52,53]. The *o*NBS group was removed by treatment with thiophenol and DBU in DMF [52]. *N*-Farnesyl peptide resin **6** was cleaved with concomitant removal of the side chain protection using a cocktail of 95:2.5:2.5 TFA:TES:H_2_O. Purification by RP-HPLC and freeze-drying of the collected fractions gave *N*-farnesyl peptide **7** as white powder, which was soluble in water (Table 1).

Finally, *N*-dodecoyl-l-PDC31 (**8**) was synthesized from resin L-**1** by coupling with lauric acid using HBTU and DIEA, resin cleavage and removal of side chain protection as described above. Purification by RP-HPLC gave **8** as white powder after freeze-drying (Table 1). The poor solubility of *N*-dodecoyl-l-PDC31 (**8**) necessitated dissolving in 0.1 N HCl and freeze-drying to give the hydrochloride salt as white powder that dissolved readily in water.

Among the peptides synthesized, only peptides **4f** and **8** displayed aqueous solubility issues, which were in agreement with their calculated >0 *c*LogP values (Figure 1). Increased lipophilic character may promote peptide aggregation and decrease solubility in aqueous solvent. The potential to aggregate may increase due to the amphiphilic character of the *N*-alkyl PDC-31 analogs which possess hydrophobic and charged residues respectively at the *N*- and *C*-terminals. For biological assays, solubility issues were surmounted by forming salts and using small amounts of DMSO. The influence of their amphiphilic character during purification by RP-HPLC and double alkylation from the reductive amination sequence may account for the lower isolated yields of peptides **4**, **7** and **8**.

### 2.2. Biology

**Figure 2:**
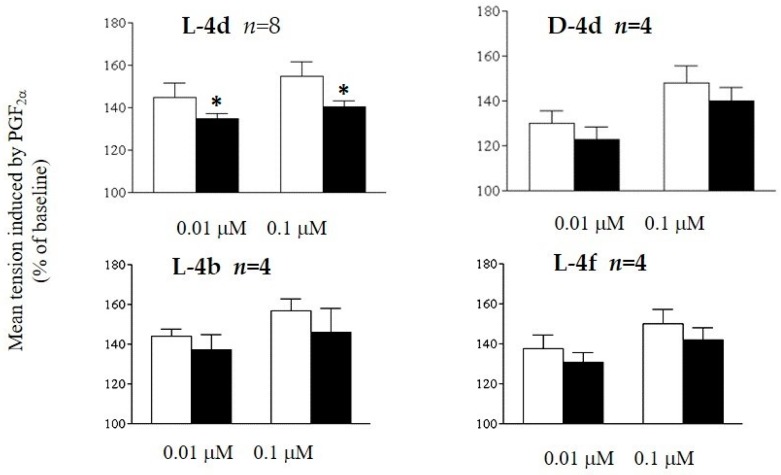
Mean tension induced by PGF_α_ on murine myometrial tissue alone (white bar) and in the presence of peptide at two different concentrations. * *p <* 0.05 compared with vehicle-treated.

All the peptides were initially tested in an ex vivo mouse myometrial tissue contraction assay. Contractile activity was measured on tissue that was harvested post-partum and treated with prostaglandin F_2α_ (PGF_α_) alone (control) or in the presence of peptides l-**4b**-**4g**, l-**7**, and l-**8**. Among all the peptides tested, only *N*-dodecyl-l-PDC31 (l-**4d**) exhibited a significant reduction of contractions (Figure 2). Peptides d-**4d**, l-**4b** and l-**4f**, all exhibited trends suggesting protective effects against PGF_2α_-induced contractions. Mean tension induced by KCl (as a positive control) was not affected by l-**4d**.

**Figure 3:**
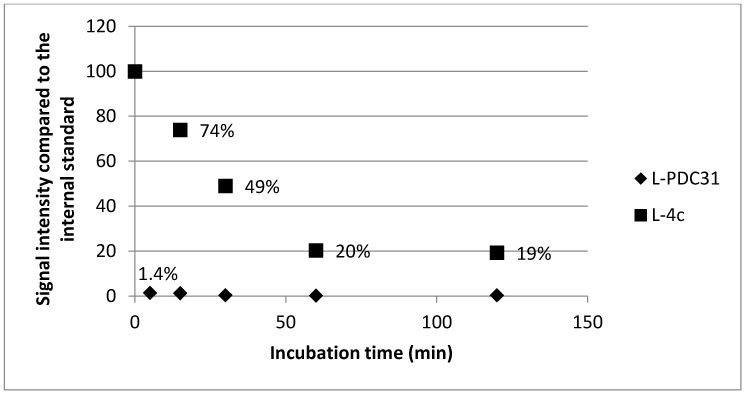
Intensity of MS signal of l-PDC31 and l-**4d** compared to the internal standard **l-4f** as a function of incubation time in mouse plasma.

Peptide l-**4d** was further examined for stability in serum and compared to l-PDC31. The two peptides were incubated at 37 °C in mouse plasma. Aliquots were removed at different time intervals and analyzed by mass spectrometry (MS/MS) to detect remaining amount of peptide (Figure 3). Although l-PDC31 was completely degraded within 5 minutes, the *N*-alkylated peptide l-**4d** exhibited a half-life of about 30 minutes and was still present after 2 hours of incubation. The *N*-dodecyl group substantially improved the stability of the peptide in plasma, likely by conferring resistance to proteolysis.

**Figure 4:**
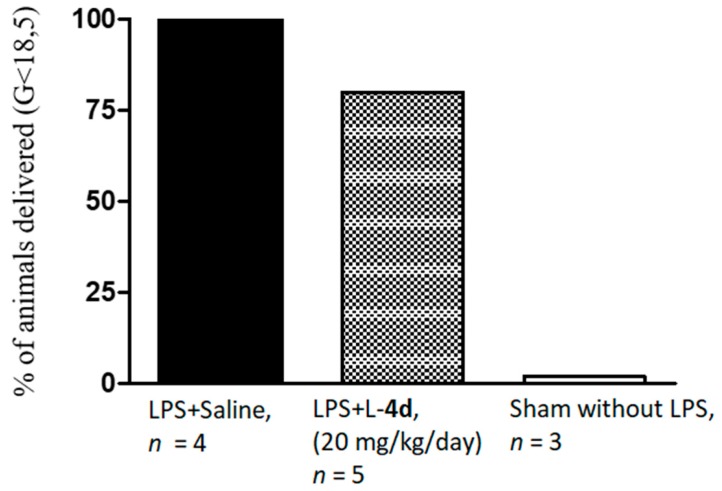
In LPS-induced mouse PTB assay, peptide L-**4d** exhibited no significant ability to delay labor.

The ability of peptide l-**4d** to delay labor in vivo was next tested in mice near term and treated with lipopolysaccharide (LPS) *Escherichia coli* endotoxin, as a pathogen associated molecular pattern, which is known to promote a general inflammatory state resulting in prostaglandin synthesis and induced premature delivery. Following LPS injection into mice (*n* = 4–5) at gestational day 16, all the animals tested delivered within 12−24 h. In contrast to PDC-31 and small molecule mimics, which have previously been shown to delay labor in the PTB model [54,55,56], L-**4d** was unable to exhibit a statistically significant reduction in the time of delivery (Figure 4).

## 3. Discussion

The d-peptide PDC-31 was previously shown to reduce both the strength and length of spontaneous and PGF2α-induced contractions in postpartum mice myometrium [18]. Examination of peptide conjugates l-**4b-g,** l-**7** and l-**8** for ability to inhibit PGF2α-induced contractions in the same assay demonstrated that *N*-dodecyl peptide l-**4d** was able to reduce uterine contractions induced by PGF2α in mice myometrial tissue [57]. Moreover, addition of the dodecyl chain enhanced plasma stability. In contrast, the parent L-peptide, l-PDC-31, did not show any inhibitory activity against PGF2α-induced contractions and was rapidly degraded in plasma. Myometrial tissue is rich in proteases [58], which may degrade and render the linear l-peptide inactive. The related *N*-hexanyl and *N*-octadecanyl peptides l-**4b** and l-**4f** exhibited trends in the myometrium contraction assay which may suggest an inhibitory potential, but the results were not statistically significant (Figure 2). Octadecanyl peptide **4f** was poorly soluble in water and needed slight amounts of DMSO to be tested, which, in turn, could explain the poor activity in the ex vivo assay. Neither PEGylated peptide l-**4g** nor *N*-farnesyl peptide l-**7** exhibited activity. Similarly, *N*-dodecanoyl peptide l-**8** and its HCl salt were both inactive. The latter result was consistent with the inactivity of *N*-acetyl-PDC31 [19] and highlighted the importance of a basic amine terminal for peptide activity.

In addition to *N*-alkylation rendering the L-peptide more stable to degradation in plasma, the lipid chain may compliment the hydrophobic (Ile-Leu-Gly) *N*-terminus to anchor the peptide in cell membranes and enhance activity and affinity for the membrane-bound receptor. To explore the latter hypothesis, the dodecyl chain was added onto PDC-31 to give D-**4d**. Although it exhibited a tendency similar to the activity of l-**4d**, d-**4d** affected myometrial contraction with a lower potency than the unmodified d-peptide, PDC-31 [18]. Thus, the alkyl chain appears to interfere with receptor binding [18] and likely exhibits its effect by enhancing resistance to proteolytic degradation.

Although activity was maintained and plasma stability was improved by the addition of a dodecyl chain to l-PDC-31, l-**4d** failed to prolong labor in vivo in the LPS-induced mouse model. The high lipophilicity of the dodecyl chain may inhibit delivery to the target receptor; instead, l-**4d** may be trapped in adipose tissue or cellular membranes, an effect that has been reported in other lipid-peptide conjugates [59].

## 4. Materials and Methods

### 4.1. General

Unless otherwise noted, synthetic grade reagents were obtained from commercial sources and used without further purification. Anhydrous solvents (THF, DMF and DCM) were obtained by passage through solvent filtration systems (GlassContour, Irvine, CA). Solution phase reactions were performed under argon atmosphere. Dodecanal and farnesol were purchased from commercial sources and used without further purification. Hexanal [60], undecanal [61], tridecanal [62], octadecanal [63], and 2,5,8,11-tetraoxatridecan-13-al [64] all were obtained by oxidation of the corresponding primary alcohol and exhibited characterization consistent with the published literature. Final purity assessments were made based on an analytical RP-HPLC-MS system from Agilent Technologies Inc. (Santa Clara, CA, USA) using the following columns and conditions: Phenomenex Synergi Polar-RP (Phenomenex, Torrance, CA, USA), 4,6 mm × 150 mm, 4 µm, UV detection (Agilent Technologies Inc., Santa Clara, CA, USA) at 250 nm or 280 nm, 5–50% MeCN with 0.1% formic acid (FA) in water (0.1% FA) over 20 min at 0.5 mL/min, or 30–90% MeOH (0.1% FA) in water (0.1% FA) over 20 min at 0.5 mL/min; Phenomenex C18 4.6 mm × 50 mm, 4 µM, UV detection at 280 nm, 10–90% MeCN (0.1% FA) in water (0.1% FA) over 15 min at 0.5 mL/min, or 10–90% MeOH (0.1% FA) in water (0.1% FA) over 15 min at 0.5 mL/min. Analytical RP-HPLC (Agilent Technologies Inc., Santa Clara, CA, USA) was performed on a Phenomenex SunFire C18 column, 2.1 × 50 mm, 3.5 um, with UV detection at 254 or 280 nm, using a gradient of 10–90% water (0.1% FA) in acetonitrile (0.1% FA) over 10 minutes at 0.35 mL/min. Accurate mass measurements were performed on a LC-MS-TOF instrument from Agilent Technologies Inc. (Santa Clara, CA, USA) using the positive electrospray mode for high resolution MS (HRMS) analysis, which was performed at the Centre regional de spectrométrie de masse de l’Université de Montréal (Montréal, PQ, Canada). Protonated ions [M + H]^+^ were used for empirical formula confirmation.

### 4.2. Chemistry

#### 4.2.1. Resin Loading and Capping

2-Chlorotrityl chloride resin (GLS Biochem, 1g, 0.8 mmol) swollen in DMF was treated with Fmoc-l-Lys(Boc)-OH (1.124 g, 2.4 mmol), HBTU (910 mg, 2.4 mmol) and diisopropyl ethylamine (835 µL, 4.8 mmol) in 10 mL of DMF. The reaction mixture was agitated on an orbital shaker for 3 h, the resin was filtered, and the coupling was repeated. The resin was filtered and washed with DMF (3 × 15 mL), isopropyl alcohol (3 × 15 mL) and DCM (3 × 15 mL). The resin was swollen in DMF (25 mL), treated with diisopropyl ethylamine (835 µL, 4.8 mmol) in 10 mL methanol, and agitated for 2 h. The resin was filtered, washed with DMF (3 × 15 mL), isopropyl alcohol (3 × 15 mL) and DCM (3 × 15 mL) and dried under vacuum. The efficiency of the coupling was determined after Fmoc removal and analysis of the dibenzofulvene adduct using UV analysis [65].

#### 4.2.2. Automated Peptide Synthesis

Peptide synthesis was performed using a CEM Liberty microwave peptide synthesizer (CEM, Matthews, NC, USA), employing the following protected amino acids: Fmoc-Lys(Boc)-OH, Fmoc-Tyr(^t^Bu)-OH, Fmoc-Asp(^t^Bu)-OH, Fmoc-Cit-OH, Fmoc-His(Tr)-OH, Fmoc-Gly-OH, Fmoc-Leu-OH, and Fmoc-Ile-OH. The Fmoc group was removed by using two treatments with 20% piperidine in DMF with microwave irradiation at 75 °C (35W) at first for 30 sec, and then for 180 sec in the second treatment. After the resin was washed (4 × 25 mL DMF), couplings were performed in DMF with N^α^-Fmoc amino acid (300 mol%, 0.2 M), HBTU (300 mol%, 0.5 M) and DIEA (600 mol%, 2 M). Typically, the N^α^-Fmoc amino acid solution (30 mL, 6 mmol) was added to the resin, followed by the HBTU (12 mL, 6 mmol) and DIEA (6 mL, 12 mmol) solutions. For all the amino acids except histidine, the coupling cycle was performed with irradiation at 75 °C (25W) for 300 sec. In the case of histidine, the coupling cycle was performed without irradiation initially at 50 °C for 120 sec, followed by irradiation at 50 °C (25W) for 240 sec. The resin was washed (3 × 25 mL DMF) prior to each coupling step. All the residues were coupled using a single reaction cycle. Upon completion of the sequence, the resin was washed with 10 mL of DCM and transferred to the receiving flask using 3 × 10 mL volumes of DCM. The final Fmoc group removal was done manually by swelling the resin in a solution of 20% piperidine in DMF (25 mL) and agitating for 30 min. The resin was filtered and washed with DMF (3 × 25 mL), isopropanol (3 × 25 mL), dichloromethane (3 × 25 mL) and ethyl ether (1 × 30 mL), dried under vacuum and stored at −20 °C.

#### 4.2.3. Peptide Cleavage and Purification

After removal of the terminal Fmoc group as described above, the resin was treated with a cleavage cocktail of TFA:TES:H_2_O (95:2,5:2,5) for 2 h and filtered. The resin was washed once with TFA and once with DCM. The washing and filtrate were combined and evaporated to an oil, which was washed with cold ether and purified by RP-HPLC using a Synergi RP-Polar C18, 150 mm × 21.2 mm column, 10 mL/min flow rate, detection at 254 nm or 280 nm, and 5–90% MeCN (0.1% FA) in water (0.1% FA) as eluent over 40 min. Collected fractions were freeze-dried to afford pure peptides (Table 1).

*H-Ile-Leu-Gly-His-Cit-Asp-Tyr-Lys-OH* (l-**PDC31**): Resin l-**1** (221 mg, 0.07 mmol) was agitated with a mixture of 15 mL of TFA:TES:H_2_O (95:2.5:2.5) for 2 h, filtered and washed with TFA (1 × 5 mL) and DCM (1 × 5 mL). The filtrate and washings were combined and evaporated to an oil, which was washed with cold ether and purified by RP-HPLC using 5–50% MeCN (0.1% FA) in H_2_O (0.1% FA). Free-drying of the collected fractions gave l-**PDC31** as white solid (5 mg).

*N-Hexanyl-Ile-Leu-Gly-His-Cit-Asp-Tyr-Lys-OH* (l-**4b**): A dry sample of 912 mg (0.3 mmol) of Ile-Leu-Gly-His(Tr)-Cit-Asp(tBu)-Tyr(tBu)-Lys(Boc) resin L-**1** in a plastic syringe tube equipped with Teflon™ filter, stopcock and stopper was swollen in THF (15 mL) and treated with hexanal (613 mg, 6 mmol) agitated on an orbital shaker for 18 h at room temperature, filtered and washed with DMF (3 × 15 mL), dichloromethane (3 × 15 mL) and THF (1 × 20 mL). The resin was swollen in THF (15 mL), treated with sodium cyanoborohydride (150 mg, 2.4 mmol), agitated on an orbital shaker for 18 h, filtered and washed with DMF (3 × 15 mL), isopropanol (3 × 15 mL) and dichloromethane (3 × 15 mL). The resin was exposed to a mixture of TFA:TES:H_2_O (95:2.5:2.5), stirred for 2 h, filtered and washed with TFA (1 × 5 mL) and DCM (1 × 5 mL). The filtrate and washing were combined, evaporated to a residue, which was washed with cold ether and purified by RP-HPLC using 5–50% MeCN (0.1% FA)/H_2_O (0.1% FA). Freeze drying of the collected fractions gave *n*-hexyl peptide l-**4b** as a white solid (1 mg).

*N-Undecanyl-Ile-Leu-Gly-His-Cit-Asp-Tyr-Lys-OH* (l-**4c**): The protocol for the synthesis of hexanyl peptide L-**4b** described above was adapted for undecanal. The obtained oil was purified by RP-HPLC using 5–50% MeCN (0.1% FA)/H_2_O (0.1% FA). Freeze-drying of the collected fractions gave l-**4c** as white solid (8 mg).

*N*-*Dodecyl-Ile-Leu-Gly-His-Cit-Asp-Tyr-Lys-OH* (l-**4d**): The protocol for the synthesis of hexanyl peptide l-**4b** described above was adapted for dodecanal. The obtained oil was purified by RP-HPLC using 5–50% MeCN (0.1% FA)/H_2_O (0.1% FA). Freeze-drying of the collected fractions gave l-**4d** as white solid (2 mg).

*N*-*Dodecyl-d-Ile-d-Leu-Gly-d-His-d-Cit-d-Asp-d-Tyr- d-Lys-OH* (d-**4d**): The protocol for the synthesis of hexanyl peptide L-**4b** described above was adapted for dodecanal on H- d -Ile- d -Leu-Gly- d -His(Tr)- d -Cit-d-Asp(tBu)-d-Tyr(tBu)- d -Lys(Boc) resin **D-1**. The oil was purified by RP-HPLC using 5–50% MeCN (0.1% FA)/H_2_O (0.1% FA). Freeze-drying of the collected fractions provided d-**4d** as a white solid (4 mg).

*N*-*Tridecanyl-Ile-Leu-Gly-His-Cit-Asp-Tyr-Lys-OH* (l-**4e**): The protocol for the synthesis of hexanyl peptide L-**4b** described above was adapted for tridecanal. The oil was purified by RP-HPLC using 5–50% MeCN (0.1%FA)/H_2_O (0.1% FA). Freeze-drying of the collected fractions gave octadecanyl peptide l-**4e** as white solid (3 mg).

*N*-*Octadecanyl-Ile-Leu-Gly-His-Cit-Asp-Tyr-Lys-OH* (l-**4f**): The protocol for the synthesis of hexanyl peptide l-**4b** described above was adapted for octadecanal. The oil was purified by RP-HPLC using 5–50% MeCN (0.1%FA)/H_2_O (0.1% FA). Freeze-drying of the collected fractions gave octadecanyl peptide l -**4f** as white solid (1 mg).

*N-2,7,8,11-tetraoxytetradecane-Ile-Leu-Gly-His-Cit-Asp-Tyr-Lys-OH* (l-**4g**): The protocol for the synthesis of hexanyl peptide L-**4b** described above was adapted for 2,5,8,11-tetraoxatridecan-13-al. The resulting oil was purified by RP-HPLC (5–50% MeCN (0.1% FA)/H_2_O + 0.1% FA). Freeze-drying of the collected fractions gave L-**4g** as white solid (10 mg).

*N*-*o*-*Nitrophenylsulfonyl-Ile-Leu-Gly-His(Tr)-Cit-Asp(tBu)-Tyr(tBu)-Lys(Boc) Resin***5**: Resin l-**1** (917 mg, 0.3 mmol) was swollen in DMF, treated with diisopropyl ethylamine (0.742 µL, 1.68 mmol) and 2-nitrobenzenesulfonyl chloride (190 mg, 0.85 mmol) in 10 mL of DMF, agitated on an orbital shaker for 48 h at room temperature, filtered, and washed with DMF (3 × 15 mL), isopropyl alcohol (3 × 15 mL) and DCM (3 × 15 mL). An aliquot of resin was cleaved with 1 mL of a cocktail of TFA:TES:H_2_O (95:2.5:2.5), filtered and evaporated to a residue that was examined by LC-MS analysis, which showed complete conversion [Rt = 4.33 min, C_18_, 10–90% MeCN (0.1% FA) in water (0.1% FA) over 15 min at 0.5 mL/min.]; *m*/*z* = [M]^+^ 1187.5. The resin was used in the next step without further treatment.

*N*-*o-(NBS)-N-farnesyl-Ile-Leu-Gly-His(Tr)-Cit-Asp(tBu)-Tyr(tBu)-Lys(Boc) Resin***6**: The Mitsunobu reaction was performed using conditions previously reported. ^53a^ In brief, resin **5** (0.3 mmol) was swollen in THF, treated with triphenylphosphine (441 mg, 1.68 mmol) and diisopropyl azodicarboxylate (DIAD, 330 µL, 1.68 mmol) in 10 mL of THF, agitated for 10 min on an orbital shaker, treated with farnesol (420 µL, 1.68 mmol) and agitated at room temperature for 48 h. The resin was washed with DMF (3 × 15 mL), isopropanol (3 × 15 mL) and DCM (3 × 15 mL). An aliquot of resin was cleaved with 1 mL of a cocktail of TFA:TES:H_2_O (95:2.5:2.5), filtered and evaporated to a residue, that was examined by LC-MS analysis, which showed 65% conversion [Rt = 5.68 min, C_18_, 10–90% MeCN (0.1% FA) in water (0.1% FA) over 15 min at 0.5 mL/min]; *m*/*z* = [M + 2H]^2+^ 696.5. The resin was used in the next step without further purification.

*N*-*Farnesyl-Ile-Leu-Gly-His-Cit-Asp-Tyr-Lys-OH* (l-**7**): Resin 6 was swollen in 15 mL of DMF, treated with thiophenol (300 µL, 2.93 mmol) and DBU (500 µL, 3.34 mmol), agitated on an orbital shaker for 6 h, filtered and washed with DMF (3 × 15 mL), isopropanol (3 × 15 mL) and DCM (3 × 15 mL). The resin was treated with 10 mL of a cocktail of TFA:TES:H_2_O (95:2.5:2.5), agitated for 2 h, filtered, and washed with TFA (1 × 5 mL) and DCM (1 × 5 mL). The filtrate and washings were combined and evaporated to an oil, which was washed with cold ether and purified by RP-HPLC using 5–50% MeCN (0.1% FA)/H_2_O (0.1% FA). Freeze-drying of the collected fractions provided farnesyl peptide l-**7** as white solid (9 mg).

*N*-*Dodecanoyl-Ile-Leu-Gly-His-Cit-Asp-Tyr-Lys-OH* (l-**8**): A dry sample of resin l-**1** (3.04 g, 1 mmol) was swollen in THF (15 mL) in a plastic syringe tube equipped with Teflon™ filter, stopcock and stopper, and treated with lauric acid (613 mg, 3 mmol), DIEA (1.05 mL, 6 mmol) and HBTU (1.14 g, 3mmol), agitated on an orbital shaker for 3 h at room temperature, filtered and washed with DMF (3 × 25 mL), isopropanol (3 × 25 mL), dichloromethane (3 × 25 mL) and THF (1 × 30 mL). The resin was exposed to a mixture of TFA:TES:H_2_O (95:2.5:2.5), stirred for 2h, filtered and washed with TFA (1 × 10 mL) and DCM (1 × 10 mL). The filtrate and washing were combined, evaporated to a residue, which was washed with cold ether, and purified by RP-HPLC using 5–50% MeCN (0.1% FA)/H_2_O (0.1% FA). Freeze drying of the collected fractions gave l-**8** as a white solid (159 mg).

### 4.3. Biology

#### 4.3.1. Serum Stability Study

Serum was warmed and kept on ice throughout the procedure. Two blanks were prepared by adding 5 µL of DMSO to 95 µL of serum. These were incubated for 2 h and submitted to the same treatment as the other samples. Two series of vials containing 95 µL of serum were prepared and spiked with either 5 µL of l-**PDC31** [0.4 mM] or l-**4d** [0.4 mM], and incubated for 120, 60, 30, 15, 10 and 5 min, respectively, after which 100 µL of 4% phosphoric acid was added. Two vials were also spiked with 5 µL l-PDC31 and L-**4d**, respectively, and immediately treated with phosphoric acid (time = 0 min). The vials were incubated for 5 min at 95 °C, cooled on ice for 2 min, treated with 600 µL of cold MeCN containing l-**4f** (0.02 mM) as internal standard, and incubated at –20 °C overnight. After the incubation period, the vials were vortexed for 2 min and centrifuged at 4 °C for 15 min. The supernatant was transferred into LC-MS vials and analyzed.

Results were expressed as: (ANISAN0IS0)∗100

AN and IS refer, respectively, to the analyte and internal standard MS signals. Both values are compared with initial injection at which time AN_0_ and IS_0_ were not incubated.

#### 4.3.2. Ex Vivo Study

Pregnant CD-1 mice (16-17 days gestation, term 19 days) were obtained from Charles River Inc. and used according to a protocol of the Animal Care Committee of Hôpital Sainte-Justine according to the principles of the Guide for the Care and Use of Experimental Animals of the Canadian Council on Animal Care. The animals were maintained on standard laboratory chow under a 12:12 light:dark cycle and allowed free access to chow and water.

The uterus from the CD-1 mouse was obtained from the animal immediately after term delivery under anesthesia (2.5% isoflurane). Briefly, a midline abdominal incision was made and the uterine horns were rapidly excised, cleansed carefully of the surrounding connective tissues, and removed. Longitudinal myometrial strips (2 to 3 mm wide and 1 cm long) were dissected free from the uterus and mounted isometrically in organ tissue baths. The initial tension was set at 2 g. The tissue baths contained 20 mL of Krebs buffer of the following composition (in mM): 118 NaCl, 4.7 KCl, 2.5 CaCl_2_, 0.9 MgSO_4_, 1 KH_2_PO_4_, 11.1 glucose, and 23 NaHCO_3_ (pH 7.4). The buffer was equilibrated with 95% oxygen/5% carbon dioxide at 37 °C. Isometric tension was measured by a force transducer and recorded by a BIOPAC data acquisition system (BIOPAC MP150, Montreal, PQ, Canada). Experiments were begun after 1 h equilibration. Mean tension of spontaneous contractions was measured using a BIOPAC digital polygraph system (AcqKnowledge); the same parameters were also determined after addition of PGF_2α_ in the presence or the absence of a 20 min-pretreatment with different FP inhibitors. At the start of each experiment, mean tension of spontaneous myometrial contractions was considered as a reference response. Changes in mean tension (g) were expressed as percentages of the initial reference response (% of baseline).

At the start of each experiments, mean tension of spontaneous myometrial contractions was considered as a reference response. Increase in mean tension (%) was expressed as percentages of (X/Y)-100, where X is the change in mean tension (g) induced by PGF_2α_ and Y is the initial reference response (g). Data are representative of 4–6 experiments per treated group. All results are expressed as means ± SEM and were compared by Independent *t*-tests. Statistical tests were performed with GraphPad Prism 4.3 software and *p* < 0.05 was considered statistically significant.

#### 4.3.3. In Vivo Study

Timed-pregnant CD-1 mice at 16 days gestation (normal term is 19.2 days) were anesthetized with isoflurane (2%). Primed osmotic pumps (Alzet pump, Alzet, Cupertino, CA) containing either saline (*n* = 4) or compound l-**4d** (20 mg/day/animal, *n* = 5) were respectively subcutaneously implanted on the backs of the animals; infusion of peptide was immediately preceded by bolus injection of peptide (0.1 mg/animal intraperitoneally). Within 15 min after placement of the pumps, animals were injected with lipopolysaccharide (LPS) *Escherichia coli* endotoxin (10 μg/animal intraperitoneally) to mimic the inflammatory/infectious component of human preterm labor. Animals were inspected every hour for the first 18 h and every 2 h thereafter to document the timing of birth. Results are expressed as percentages of animals delivered following the injection of LPS [57]. All the experiments were approved by the Animal Care Committee of Centre Hospitalier Universitaire Sainte-Justine (Montreal, QC, Canada).

## 5. Conclusions

Herein, we report effective solid-phase methods for installing alkyl, PEG, farnesyl and alkanoyl *N*-terminal grafts onto small peptides. Evaluation of the peptide derivatives ex vivo has identified that *N*-dodecyl peptide l-**4d** exhibited a significant reduction of PGF2α-induced contractility in post-partum ex vivo assay versus vehicle. Moreover, in mouse plasma, the dodecyl chain prolonged stability of l-**4d** due likely to a protective effect against proteases. Although the strategy proved successful ex vivo and *in vitro*, *N*-dodecyl peptide l-**4d** was inactive in vivo when given by subcutaneous injection to induced mice. Considering the proof that l-PDC31 can exhibit activity when conjugated to a dodecyl chain, further research is merited to study alternative means of administration and conjugation towards the development of a cost-effective tocolytic agent for treating preterm labor.

## Figures and Tables

**Table 1:** Retention time, purity, exact mass and overall yield of peptides **4b–4g**, **7** and **8**.

Peptide	R-ILGHCitDYK R =	Retentin Time MeCN (min)	Retentin Time MeOH (min)	Purity (%)	[M + H]^+^ Calc.	[M + H]^+^ Found	TotalYield (%)
**l** **-PDC31**	H-	10.8 ^a^	9.9 ^b^	>97	1002.5367	1002.5366	7.7
**4b**	H_3_C(CH_2_)_5_-	9.8 ^a^	9.7 ^b^	>92	1086.6306	1086.633	0.3
**4c**	H_3_C(CH_2_)_10_-	11.1 ^a^	11.9 ^b^	>89	1158.7089	1158.7034	1.4
**l** **-4d**	H_3_C(CH_2_)_11_-	12.6 ^c^	13.4 ^d^	>95	1170.7245	1170.7251	0.7
**d** **-4d**	H_3_C(CH_2_)_11_-	13.0 ^a^	13.1 ^b^	>94	1170.7245	1170.7238	1.2
**4e**	H_3_C(CH_2_)_12_-	13.9 ^a^	14.2 ^b^	>99	1184.7402	1084.7387	0.4
**4f**	H_3_C(CH_2_)_15_-	15.0 ^a^	15.1 ^b^	>94	627.9128 ([M + 2H]^2+^)	627.9105 ([M + 2H]^2+^)	0.3
**4g**	H_3_C[O(CH_2_)_2_]_4_-	10.0 ^a^	9.6 ^b^	>86	1192.6572	1192.6605	2.9
**7**	Farnesyl-	10.0 ^a^	13.3 ^b^	>89	1206.7245	1206.7263	2.5
**8**	H_3_C(CH_2_)_10_CO-	7.1^c^	10.2 ^d^	>99	1184.7038	1184.7061	15.0

^a^ Polar-RP, 5–50% MeCN with 0.1% formic acid (FA) in water (0.1% FA) over 20 min at 0.5 mL/min. ^b^ Polar-RP 30–90% MeOH (0.1% FA) in water (0.1% FA) over 20 min at 0.5mL/min. ^c^ C_18_, 10–90% MeCN (0.1% FA) in water (0.1% FA) over 15 min at 0.5 mL/min. ^d^ C_18_, 10–90% MeOH (0.1% FA) in water (0.1% FA) over 15 min at 0.5 mL/min.
